# Cost-utility, cost-effectiveness, and budget impact of Internet-based cognitive behavioral therapy for breast cancer survivors with treatment-induced menopausal symptoms

**DOI:** 10.1007/s10549-019-05410-w

**Published:** 2019-08-26

**Authors:** Joost G. E. Verbeek, Vera Atema, Janne C. Mewes, Marieke van Leeuwen, Hester S. A. Oldenburg, Marc van Beurden, Myra S. Hunter, Wim H. van Harten, Neil K. Aaronson, Valesca P. Retèl

**Affiliations:** 1 Division of Psychosocial Research and Epidemiology, The Netherlands Cancer Institute, P.O. Box 90203, 1006 BE Amsterdam, The Netherlands; 2 Department of Health Technology and Services Research, Unveristy of Twente, Enschede, The Netherlands; 3 Panaxea, Amsterdam, The Netherlands; 4 Department of Surgical Oncology, The Netherlands Cancer Institute, Amsterdam, The Netherlands; 5 Department of Gynecology, The Netherlands Cancer Institute, Amsterdam, The Netherlands; 6 Department of Psychology, Institute of Psychiatry, Psychology and Neuroscience, Kings College London, London, UK

**Keywords:** Cost-effectiveness, Budget impact, Menopause, Breast cancer, Cognitive behavioral therapy, Internet-based

## Abstract

**Purpose:**

Internet-based cognitive behavioral therapy (iCBT), with and without therapist support, is effective in reducing treatment-induced menopausal symptoms and perceived impact of hot flushes and night sweats (HF/NS) in breast cancer survivors. The aim of the current study was to evaluate the cost-utility, cost-effectiveness, and budget impact of both iCBT formats compared to a waiting list control group from the Dutch healthcare perspective.

**Methods:**

A Markov model was constructed with a 5-year time horizon. Costs and health outcomes were measured alongside a randomized controlled clinical trial and included quality-adjusted life years (QALYs), overall levels of menopausal symptoms, and perceived impact of HF/NS. Uncertainty was examined using probabilistic and deterministic sensitivity analyses, together with a scenario analysis incorporating a different perspective.

**Results:**

iCBT was slightly more expensive than the waiting list control, but also more effective, resulting in incremental cost-utility ratios of €23,331/QALY and €11,277/QALY for the guided and self-managed formats, respectively. A significant reduction in overall levels of menopausal symptoms or perceived impact of HF/NS resulted in incremental costs between €1460 and €1525 for the guided and €500–€753 for the self-managed format. The estimated annual budget impact for the Netherlands was €192,990 for the guided and €74,592 for the self-managed format.

**Conclusion:**

Based on the current trial data, the results indicate that both guided and self-managed iCBT are cost-effective with a willingness-to-pay threshold of well below €30,000/QALY. Additionally, self-managed iCBT is the most cost-effective strategy and has a lower impact on healthcare budgets.

## Introduction

Adjuvant treatments for breast cancer (BC), including chemotherapy, endocrine therapy, and oophorectomy can lead to treatment-induced menopausal symptoms [1, 2]. These symptoms, and in particular hot flushes and night sweats (HF/NS), negatively affect health-related quality of life (HRQL) [3–5] and cause some women to discontinue their endocrine treatments [6, 7]. Although medications such as gabapentin, clonidine, and antidepressants are moderately effective in reducing HF/NS, they are accompanied by bothersome side effects [8–11]. In contrast, cognitive behavioral therapy (CBT) programs are without side effects, are effective, and are favored by BC survivors [12–16].

CBT programs have often been delivered in group format [14–16]. However, BC survivors have reported practical and scheduling barriers to attending such group sessions [16]. Therefore, these programs have been translated into an online format [17, 18]. Our recent randomized controlled trial (RCT) comparing Internet-based CBT (iCBT), with and without therapist support, with a waiting list control group demonstrated that women allocated to iCBT experienced a greater reduction in overall levels of menopausal symptoms and perceived impact of HF/NS. Significant reductions in the frequency of HF/NS and improvement in sleep quality were also observed [19]. When asked about preferences for a specific format, only a minority of women showed a strong preference for guided (16%) or self-managed (21%) iCBT. Although the magnitude of the effects favored the guided over the self-managed iCBT group, the former is associated with higher costs due to the added therapist support.

The observed differences in effectiveness and costs between the iCBT formats and the reality of budget restrictions underscore the need for an economic evaluation to assist policymakers in deciding whether to allocate healthcare resources to this program. Moreover, it may also guide practitioners in choosing which specific format to adopt [20]. Although a previous study by Mewes et al. [21] indicated that face-to-face group-based CBT was cost-effective, it is unknown whether online-delivered CBT will lead to favorable cost-effectiveness ratios as well. Moreover, there are no studies reporting the budget impact of iCBT for treatment-induced menopausal symptoms, commonly used to estimate the impact on national, regional, or local health budget plans [22].

The objective of the current study was to evaluate the cost-utility and cost-effectiveness of guided and self-managed iCBT compared to a waiting list control group in terms of quality-adjusted life years (QALYs) and the primary clinical outcomes of the associated RCT (i.e., overall levels of menopausal symptoms and perceived impact of HF/NS), incorporating a healthcare perspective over a 5-year time period. An additional aim was to establish the estimated annual budget impact of implementing guided and/or self-managed iCBT in the Netherlands.

## Methods

### Research design and study sample

A detailed description of the design, interventions, and outcomes of the RCT is provided elsewhere [18, 19]. Briefly, from 2015 to 2017, an RCT was conducted to evaluate the efficacy of iCBT, with and without therapist support, for treatment-induced menopausal symptoms in BC survivors. Patients were recruited from 12 hospitals in the Netherlands. Upon return of the informed consent and the baseline questionnaire (T0), 254 patients were randomized to a guided iCBT group, self-managed iCBT group, or a waiting list control group. Follow-up assessments were administered at 10 weeks (T1) and 24 weeks post-randomization (T2). All institutional review boards approved the study.

### Intervention and waiting list control group

All women randomized to the intervention groups had access to a 6-week iCBT program. A strong emphasis was placed on HF/NS, but other symptoms were also addressed. Women in the guided iCBT group received an additional telephone intake and weekly online feedback from a therapist. The average time-investment per therapist was 3 h per patient. Participants allocated to the waiting list control group received usual care, which did not involve any form of care aimed at coping with menopausal symptoms.

### Measures

#### Measurement and valuation of outcomes

HRQOL was assessed using the 36-item Short Form Health Survey (SF-36) [23, 24]. To obtain utilities, scores on the eight scales were transformed into a single EQ-5D utility score using the mapping algorithm of Ara and Brazier [25]. An additional algorithm was used to verify reliability of this conversion [26]. The EQ-5D utility scores can range between 0 and 1, with higher scores indicating better health. To calculate QALYs, we multiplied the derived utility scores with years of life (mortality rates) in the relevant health states.

The menopause-specific measures included overall levels of menopausal symptoms, as assessed by the Functional Assessment of Cancer Treatment-Endocrine Symptoms (FACT-ES) [27], and the perceived impact of HF/NS as assessed by the problem rating subscale of the Hot Flush Rating Scale (HFRS) [28]. A clinically significant improvement was defined as a 0.5 standard deviation (SD) improvement for both measures [19, 27, 29, 30].

#### Measurement and valuation of costs

Costs of the iCBT program were related to the online platform and therapist support. The online costs for the guided iCBT program were dependent on the number of therapists, irrespective of the number of patients, whereas the number of patients determined the online costs for the self-managed format. Valuations of the resources used were based on cost information provided by two potential providers of iCBT in the Netherlands, and invoices obtained during the RCT (e.g., hourly therapist rates).

Direct healthcare costs were measured during the RCT by the Dutch iMTA Medical Consumption Questionnaire (*i*MCQ) [31]. Healthcare costs included the average number of visits to a range of healthcare providers (general practitioner, medical specialist, psychologist/psychiatrist, social worker, physiotherapist, lymphedema therapist, dietitian, and a practitioner of complementary alternative medicine). Valuation of visits to healthcare providers was based on the Dutch costing manual for economic evaluations [32, 33]. Mean per patient resource use and valuation can be found in Table 1. Both types of costs (intervention and healthcare utilization) were applied in the healthcare perspective.

**Table 1:** Input cost parameters in the MARKOV model

Parameters	Mean	Standard error	Distribution	Sources
Utilities				
Menopausal symptoms	0.83	0.013	Beta	[19]
Reduction in menopausal symptoms	0.85	0.017	Beta	[19]
Recurrence	0.73	0.020	Beta	[54]
Transition probabilities				
Menopausal symptoms to reduction in menopausal symptoms (guided iCBT)	0.44	–	Dirichlet	[19]
Menopausal symptoms to reduction in menopausal symptoms (self-managed iCBT)	0.39	–	Dirichlet	[19]
Menopausal symptoms to reduction in menopausal symptoms (waitlist control group iCBT)	0.23	–	Dirichlet	[19]
To recurrence from either state of menopausal symptoms or reduction in menopausal symptoms	0.01	–	Beta	[35]
Recurrence to death	0.04	–	Beta	[36]
Background mortality (age 47 to 51)	0.0007–0.0012	–	Fixed	
Intervention costs^a, b^				
Online platform costs (guided iCBT)	€ 12.59	–	–	Practice
Online platform costs (self-managed iCBT)	€ 33.28	–	–	Practice
Training costs therapists	€ 9.42	–	–	Practice
Hourly rate therapist support (in total 3 h needed to support patient)	€ 135.00	–	–	Practice
Total costs guided iCBT per patient without overhead costs	€ 157.01	–	–	Practice
Total costs self-managed iCBT per patient without overhead costs	€ 33.28	–	–	Practice
Total costs guided iCBT per patient with 44% overhead costs	€ 226.09	± 20%	Gamma	Practice
Total costs self-managed iCBT per patient with 44% overhead costs	€ 47.92	± 20%	Gamma	Practice
Health care costs				
Health state: menopausal symptoms				
General practitioner	€ 48.70	± 20%	Gamma	[19, 33]
Medical specialist	€ 152.00	± 20%	Gamma	[19, 33]
Psychologist or psychiatrist	€ 35.20	± 20%	Gamma	[19, 33]
Social worker	€ 3.25	± 20%	Gamma	[19, 33]
Physiotherapist	€ 207.78	± 20%	Gamma	[19, 33]
Lymphedema therapist	€ 106.01	± 20%	Gamma	[19, 33]
Dietitian	€ 18.74	± 20%	Gamma	[19, 33]
Alternative medicine	€ 8.96	± 20%	Gamma	[19, 33]
Health state: reduction in menopausal symptoms				
General practitioner	€ 45.38	± 20%	Gamma	[19, 33]
Medical specialist	€ 129.28	± 20%	Gamma	[19, 33]
Psychologist or psychiatrist	€ 43.37	± 20%	Gamma	[19, 33]
Social worker	€ 9.47	± 20%	Gamma	[19, 33]
Physiotherapist	€ 158.05	± 20%	Gamma	[19, 33]
Lymphedema therapist	€ 93.75	± 20%	Gamma	[19, 33]
Dietitian	€ 4.99	± 20%	Gamma	[19, 33]
Alternative medicine	€ 19.11	± 20%	Gamma	[19, 33]
Health state: recurrence				
Frist year: in- and outpatient costs	€ 10,263.00	± 20%	Gamma	[55]
First year: drug costs	€ 1918.00	± 20%	Gamma	[55]
Second year: in- and outpatient costs	€ 2294.00	± 20%	Gamma	[55]
Second year: drug costs	€ 65.00	± 20%	Gamma	[55]

*iCBT* Internet-based cognitive behavioral therapy

^a^Assumption that 600 patients enroll in iCBT

^b^Online platform costs are dependent on the therapists in the guided format, whereas these costs are dependent on the number of patients in the self-managed format

### Statistical analyses

#### Markov model

We adapted a previously developed and validated Markov model in Excel (Microsoft, Redmond, WA) in accordance with the Dutch guideline for health economic evaluations and international guidelines for modelling (ISPOR-SMDM guidelines) [22, 33, 34]. A Markov model is a stochastic approach to modelling different states and the probabilities of transitions among them (Appendix A). The following four health states were defined in the current study: (1) experience of menopausal symptoms (based on inclusion criteria of the RCT); (2) reduction in menopausal symptoms; (3) cancer recurrence (local, regional or distant); and (4) death. Transition probabilities are displayed in Table 1. The transition probabilities between the first two health states were based on the percentage of women with a clinically significant improvement per trial arm on the FACT-ES as reported by Atema et al. [19]. Transition probabilities for local, regional, and distant metastases and corresponding increased mortality rate (MR), using age- and sex-specific mortality data, were based on data from Dutch registries [35, 36].

A hypothetical cohort of 1000 patients was used in the model with an average baseline age of 47, mirroring the mean age of participants at the start of the RCT. They were analyzed over ten consecutive 6-month cycles in which the first cycle reflected the costs and effects of the iCBT as derived from the RCT [19]. The 5-year time horizon corresponds to the average duration of bothersome vasomotor symptoms of menopause [37]. The transition from health state ‘menopausal symptoms’ to ‘reduction in menopausal symptoms’ derived from the trial was only applied in the first cycle of the model. All other transitions remained applicable during consecutive cycles (Appendix A).

#### Cost-utility analysis

Incremental cost-utility ratios (ICURs) of both formats of the iCBT were calculated as follows:

$${\text{ICUR}} = \frac{{\left( {{\text{Costs of the intervention group}} - {\text{costs of the waitlist control group}}} \right)}}{{\left( {{\text{QALYs of the intervention group}} - {\text{QALYs of the waitlist control group}}} \right)}}$$

The diversity of willingness-to-pay (WTP) thresholds among countries shows that there is no uniformly accepted value. However, the World Health Organization has proposed a WTP threshold of one to three times the annual GDP per capita [38, 39]. Therefore, we estimated a WTP ceiling ratio of €30,000 per QALY for this study. Effects were discounted at 1.5% and costs at 4% annually as recommended by the Dutch costing manual [33].

#### Cost-effectiveness analysis

We also performed a cost-effectiveness analysis using the principles of number needed to treat (NNT). NNT expresses how many patients, on average, need to be treated for one less adverse event or improvement of disease to be observed at a specific point in time [40, 41]. To calculate NNT and associated costs, we used the following formulas:

$${\text{NNT}} = 1/{\text{ARR }}\left( {{\text{Absolute risk reduction}}} \right)$$

$${\text{Incremental}}\,{\text{costs}}\,{\text{to}}\,{\text{treat}}\,{\text{one}}\,{\text{patient = NNT }} \times {\text{ Incremental}}\,{\text{costs}}$$

The incremental costs to treat one patient reflect the costs per person of guided or self-managed iCBT over a 5-year period multiplied by the NNT to obtain one clinically significant reduction on the FACT-ES or HFRS problem rating scale.

#### Budget impact analysis

We performed the budget impact analysis (BIA) in accordance with ISPOR principles of good practice [22, 42]. The incremental costs were calculated using the same assumptions and model that we developed for the cost-utility analysis. We then multiplied the incremental costs by the target population in the Netherlands, which we based on previous studies [35, 43–45]. We calculated that approximately 20% of the target population (3000 invasive BC cases in women aged ≤ 50 years) will start to use the iCBT program when offered in routine care, which corresponds to 600 patients per year in the Netherlands [35]. Therefore, the current BIA reflects the annual budget impact on the Dutch healthcare system.

#### Sensitivity analyses

We used probabilistic sensitivity analysis (PSA) to estimate the uncertainty of the input parameters of the model using 5000 Monte Carlo simulations. We used Dirichlet, gamma, and beta distributions to estimate the uncertainty around transition probabilities, costs, and utilities, respectively. Uncertainty surrounding the ICURs was explored by plotting bootstrapped incremental cost-utility pairs on cost-effectiveness planes (CE-planes). A summary measure of the joint uncertainty of costs and effects for different thresholds was presented using cost-effectiveness acceptability curves (CEACs). CEACs indicate the intervention’s probability of being cost-effective compared with the waiting list control group at different values of WTP. Additionally, we examined deterministic one-way sensitivity and structural uncertainty by addressing various assumptions regarding the model such as the duration of the treatment effects (from 5 to 3 years), different healthcare costs, QALYs, and intervention costs. Corresponding parameters were based on the trial data (e.g., standard errors), ranging between two extreme yet plausible values. These analyses are displayed in tornado diagrams for both guided and self-managed iCBT separately.

We also conducted a scenario analysis in which we calculated cost-utility, cost-effectiveness, and budget impact from an intervention perspective by using only the intervention costs, meaning that we did not take into account healthcare utilization (e.g., general practitioner visits).

## Results

### Costs and QALYs

Total intervention costs for guided and self-managed iCBT were €226 and €48 per patient, respectively (Table 1). At longer-term follow-up of the RCT, healthcare costs were higher in the ‘Reduction in Menopausal’ state as compared to the state ‘Menopausal Symptoms’ (Table 1). For a 5-year time horizon, total healthcare costs were €5315.55, €5118.22, and €4993.90 for guided iCBT, self-managed iCBT, and the waiting list control group, respectively (Table 3).

The average 5-year QALY score was 4.119, 4.117, and 4.106 for guided iCBT, self-managed ICB, and the waiting list control group, respectively (Table 3).

### Cost-utility analyses

**Fig. 1:**
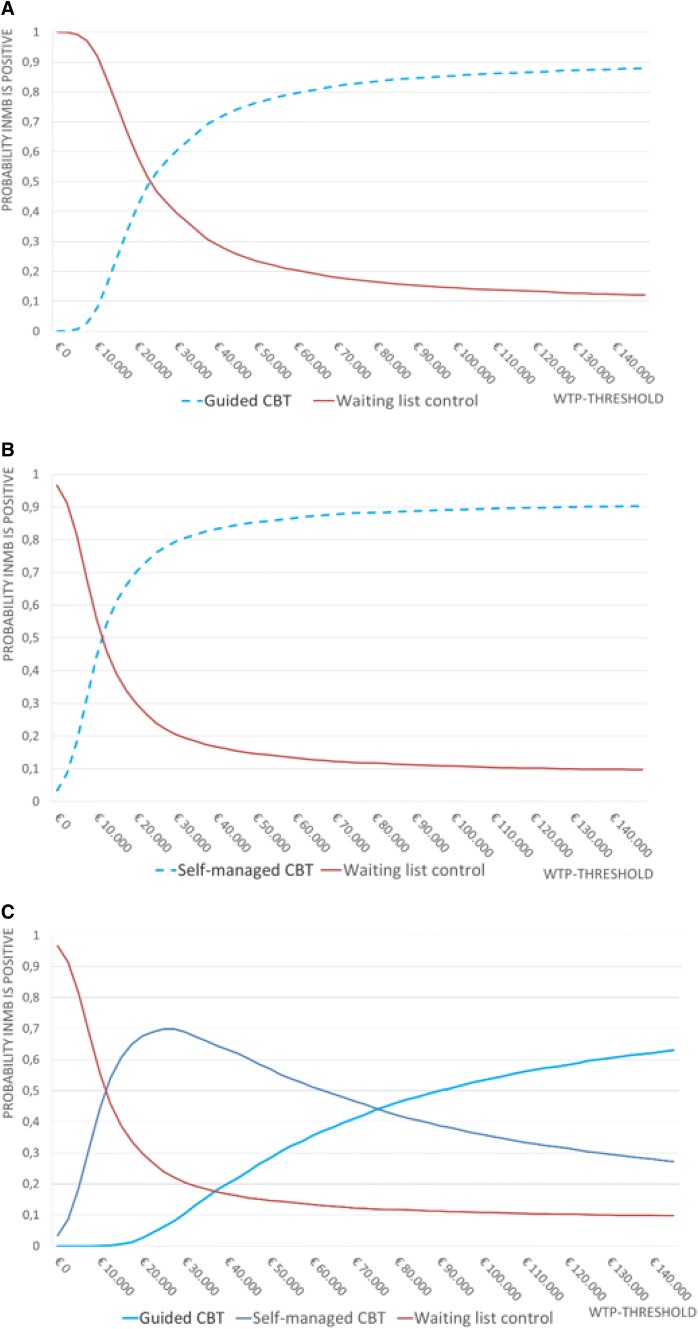
Cost-effectiveness Acceptability Curves (CEAC); presenting the probability of cost-effectiveness *for* a range of willingness-to-pay (WTP) thresholds of guided iCBT compared to waiting list control group (**a**), self-managed iCBT compared to waiting list control group (**b**), and self-managed versus guided versus waiting list control group (**c**). For a WTP threshold of 30,000 per QALY, guided and self-managed iCBT are superior over waiting list control group with a probability of 60.5% and 79.5%, respectively (**a**, **b**), and the self-managed variant is superior when comparing to both waiting list control group and guided iCBT simultaneously with a probability of 68.9% (**c**)

**Fig. 2:**
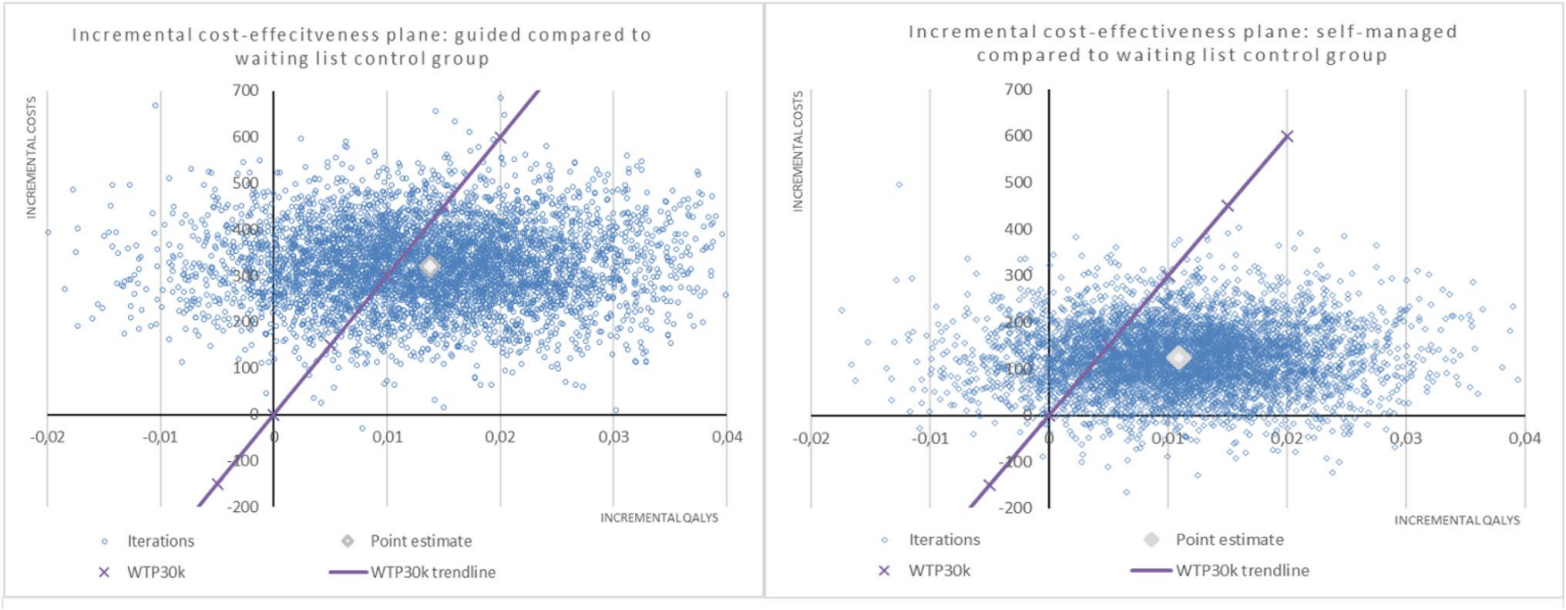
Incremental cost-effectiveness planes of the quality-adjusted life years (QALYs) per costs of the self-managed and guided iCBT intervention groups compared to a waiting list control group. The scatter plots are showing the mean differences in costs and outcomes from the data using 5000 bootstrap replicates. Ninety-two and eighty-nine percent of the dots are in the North-East quadrant of the plane for the guided and self-managed iCBT interventions, respectively. This indicates that there is a high probability that both treatments are more effective and more expensive compared to a waiting list control group

The results indicated ICURs of €23,331/QALY and €11,277/QALY for guided and self-managed iCBT, respectively (Table 2). Descriptive CEACs and iCE planes are displayed in Figs. 1 and 2, and described in the ‘sensitivity analyses’ section. For the intervention scenario, the ICURs were €16,399/QALY and €4346/QALY for guided and self-managed iCBT, respectively.

**Table 2:** Deterministic incremental cost-utility results and budget impact analyses for the base-case (FACT-ES)

	Costs	QALY	Incremental costs	Incremental QALYs	ICER	BIA^b^
Healthcare perspective						
Guided iCBT	€5315.55	4.119	€321.65	0.0138	€23,330.50	€192,990
Self-managed iCBT	€5118.22	4.117	€124.32	0.01102	€11,277.63	€74,592
Waiting list control^a^	€4993.90	4.106	n/a	n/a	n/a	n/a
Scenario analysis: intervention perspective						
Guided iCBT	€226.09	4.119	€226.09	0.0138	€16,399.45	€135,654
Self-managed iCBT	€47.92	4.117	€47.92	0.01102	€4,346.58	€28,752
Waiting list control^a^	€0.00	4.106	n/a	n/a	n/a	n/a

*BIA* budget impact analysis, *iCBT* Internet-based cognitive behavioral therapy, *ICER* incremental cost-utility ratio, *QALY* quality-adjusted life year, *n/a* not applicable

^a^Guided and self-managed interventions are compared with waiting list control group

^b^Estimated that 600 patients per year will use the intervention in the Netherlands

### Cost-effectiveness analyses

NNT calculations indicated that relatively fewer patients needed to be treated to obtain a significant reduction in menopausal symptoms (FACT-ES) in the guided iCBT format compared to the self-managed format (4.74 vs. 6.06) (Table 3). The associated costs were higher for the guided iCBT than for the self-managed iCBT (€322 vs. €124). Therefore, total incremental treatment costs to obtain a significant decrease in menopausal symptoms were smaller for the self-managed format than for the guided (€753 vs. €1,525). The same trend was observed for the intervention scenario in which incremental costs were lower for the self-managed than the guided iCBT (€290 vs. €1072).

**Table 3:** Incremental cost-effectiveness results using NNT

	Guided iCBT	Self-managed iCBT
Significant reduction on the FACT-ES^a^		
Number needed to treat (NNT)	4.74	6.06
Incremental intervention costs	€ 226.09	€ 47.92
Incremental total costs (total healthcare)	€ 321.65	€ 124.32
Total incremental costs to treat one patient (intervention perspective)	€ 1071.51	€ 290.39
Total incremental costs to treat one patient (healthcare perspective)	€ 1524.62	€ 753.38
Significant reduction on the HFRS problem rating scale^a^		
Number needed to treat (NNT)	4.54	4.02
Incremental intervention costs	€ 226.09	€ 47.92
Incremental total costs (total healthcare)	€ 321.65	€124.32
Total incremental costs to treat one patient (intervention perspective)	€ 1026.45	€ 192.64
Total incremental costs to treat one patient (healthcare perspective)	€ 1460.29	€ 499.77

*NNT* number needed to treat, *FACT*-*ES* functional assessment of cancer treatment-endocrine symptoms, *HFRS* hot flush rating scale

^a^Waiting list control group is reference category

The NNT to accomplish a significant reduction in the perceived impact of HF/NS (HFRS problem rating scale) favored the self-managed iCBT over the guided iCBT (4.54 vs. 4.02) (Table 3). Again, total incremental costs to obtain a significant decrease in the perceived impact of HF/NS were smaller for the self-managed iCBT than for the guided iCBT (€500 vs. €1460). Results for the intervention scenario indicated a similar pattern in which the incremental costs for the self-managed iCBT were lower than for the guided format (€193 vs. €1026).

### Budget impact analyses

The budget impact of treating the Dutch target population (assuming 600 patients) with guided iCBT would result in an annual net increase of €192,990 of additional expenditure from the Dutch healthcare budget to the target population. The budget impact of self-managed iCBT would result in a net increase of €74,592 (Table 2). Additionally, total health expenditure of implementing a 50/50 combination of the guided and self-managed iCBT in the Dutch setting would entail an additional cost of €133,785. Results for the intervention scenario indicated a higher 1-year net increase for the guided and the self-managed iCBT €135,654 and €28,752, respectively, and a net increase of €49,322 when implementing a combination of the guided and self-managed iCBT formats.

### Sensitivity analyses

The CEACs indicate that guided iCBT has a 60.5% probability of being cost-effective for a WTP of €30,000 (Fig. 1a). For self-managed iCBT this probability is 79.5% (Fig. 1b). Moreover, the combined CEAC indicates that self-managed iCBT has a 68.9% of being superior over guided iCBT and waiting list control with a WTP of €30,000 (Fig. 1c). For the scenario analyses (intervention perspective), the probability of cost-effectiveness for self-managed and guided iCBT is 88.8% and 72.9%, respectively, when using a WTP of €30,000 (data not shown).

The iCE planes resulted in most iterations being in the North-East quadrant (around 90%), indicating that both guided and self-managed iCBT resulted in higher costs and more QALYs (Fig. 2). Moreover, the point estimates indicated that it is likely that both self-managed and guided iCBT will be below the €30,000/QALY threshold. Results from an intervention perspective indicated a similar pattern, with an average probability of 92% of being in the North-East quadrant (data not shown).

**Fig. 3:**
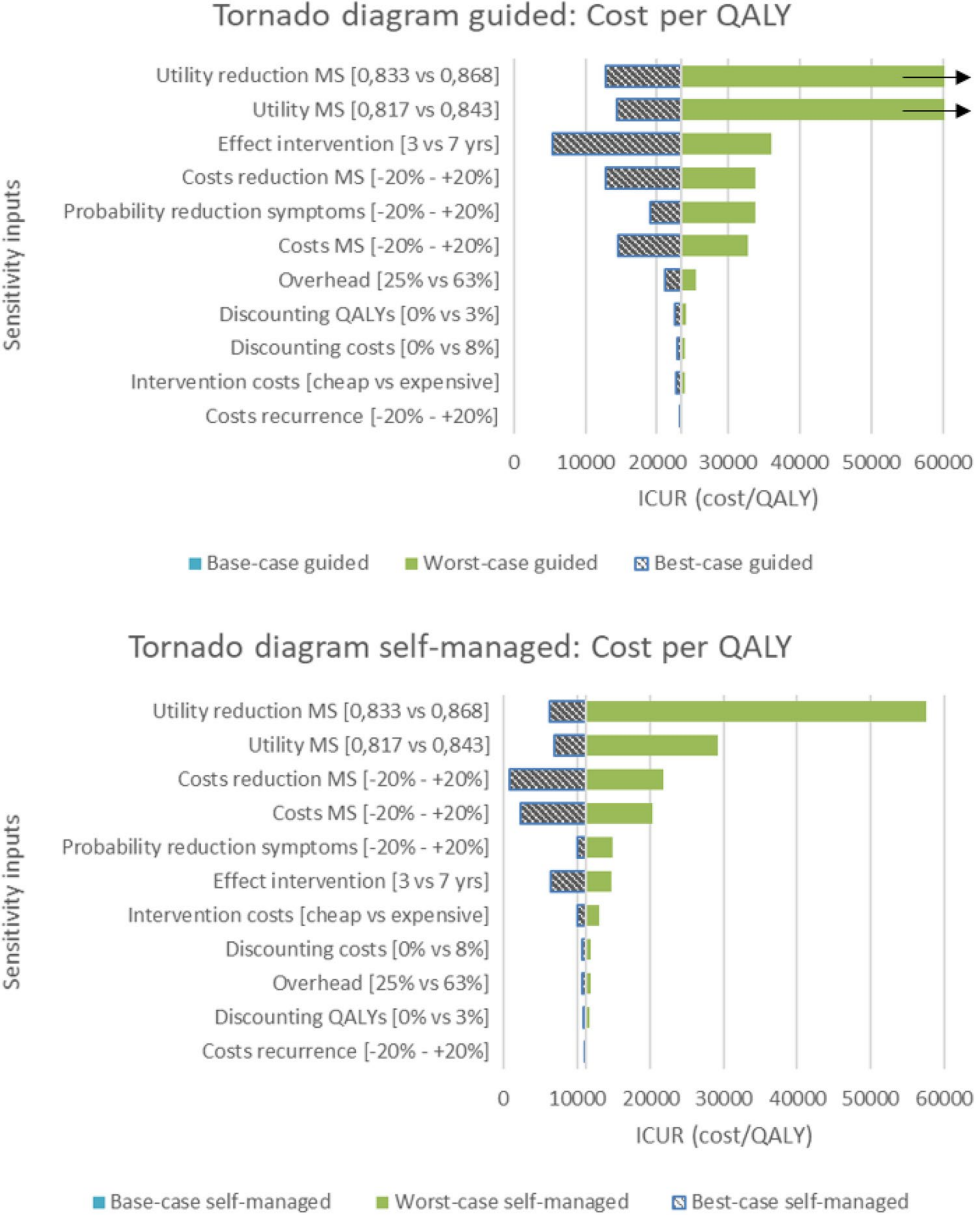
Tornado diagrams. This figure presents several univariate sensitivity analyses for both guided and self-managed iCBT. Parameters are ranked according to impact on incremental cost-utility ratio (ICUR). Results show that the utility attributed to the states ‘Reduction in Menopausal Symptoms (MS)’ and ‘Menopausal Symptoms’, the effect of the intervention lasting shmter/longer, transition probabilities, and the costs of states ‘Reduction in Menopausal Symptoms’ and ‘Menopausal Symptoms’ affect the ICUR the most. Moreover, self-managed iCBT seems to be more resistant to univariate differences in the model compared to guided iCBT

In the sensitivity analysis, the parameters of the costs and utilities associated with the states ‘Menopausal Symptoms’ and ‘Reduction in Menopausal Symptoms’ alongside the duration of intervention effects and transition probabilities showed the greatest influence on the ICER. The tornado diagrams show the impact of the uncertainty per input parameter (Fig. 3).

## Discussion

This is the first study to investigate the cost-utility, cost-effectiveness, and budget impact of iCBT to alleviate treatment-induced menopausal symptoms in BC survivors. The results show that both the guided and self-managed formats of iCBT are associated with a small gain in QALYs over a 5-year time horizon, a decrease in menopausal symptoms, and a decrease in perceived impact of HF/NS. These improvements were accompanied with an increase in costs due to additional intervention and healthcare costs. However, analyses showed that ICURs are well below the proposed international WTP threshold of €30,000/QALY for both formats [39]. The probability that the ICERs are considered acceptable ultimately depends on the willingness to pay for a clinically significant decrease in menopausal symptoms and/or perceived HF/NS. Our results indicate that, to accomplish a significant reduction in overall levels of menopausal symptoms or perceived impact of HF/NS, an investment between €1026 and €1525 for the guided and €193–€753 for the self-managed iCBT format will be necessary (the range reflects the perspective, i.e., only intervention costs, or intervention and healthcare costs). The annual Dutch budget impact (i.e., treating 600 patients) of implementing this program is estimated to be between €74,592 and €192,990 for the guided and between €28,752 and €74.592 for the self-managed iCBT. Additionally, sensitivity analyses showed that self-managed iCBT remains cost-effective (below the threshold of €30,000/QALY) for all variations in input parameters and assumptions, except when utility in the state ‘Reduction in Menopausal Symptoms’ decreases to its lower extreme value. For guided iCBT, shorter duration of intervention effects, increase in costs, decrease in utilities, and decrease in probability of obtaining a reduction in menopausal symptoms may result in unacceptable cost-effectiveness ratios, i.e., around €35,000/QALY or even higher ratios when utilities decrease unfavorably.

Compared to the economic evaluation of the group-based CBT program for alleviating menopausal symptoms in BC survivors [21], we observed similar costs per QALY outcomes for the guided format, but a reduction of more than €10,000/QALY for self-managed iCBT. We also observed higher incremental costs per clinically significant reduction in overall levels of menopausal symptoms and perceived impact of HF/NS for the guided format (± €500), and lower incremental costs per clinically significant reduction for the self-managed iCBT, when compared with the group-based CBT format [21]. This indicates that an Internet-delivered CBT program, particularly when self-managed, would be a viable alternative to face-to-face group sessions, with the added advantage of decreasing practical barriers as previously reported that hamper attendance at group sessions [16, 21]. In addition, the estimated budget impact is low in comparison with the total healthcare costs associated with the treatment of cancer in the Netherlands [46].

The increase in QALYs observed in our study and that of Mewes et al. [21] are relatively small. We believe this to be inherent to the aim of the current program, which is not primarily focused on improving overall HRQL, but rather on reducing overall levels of menopausal symptoms and perceived impact of HF/NS. When using a generic indicator of HRQL such as the SF-36, important gains in more specific domains are often missed due to the lack of responsiveness of the instrument [47], hence explaining the results from the deterministic sensitivity analysis. Therefore, cost-utility analyses should be supplemented by cost-effectiveness analyses in which the cost per condition-specific outcome are measured and taken into account in reimbursement decisions. Moreover, we encourage the development and testing of condition-specific preference-based instruments which can be used within the QALY framework [47].

Based on our findings, we would recommend implementing the iCBT program according to a stepped care approach [48] in which the self-managed program serves as the primary treatment option. Dependent on available budgets, patient preferences, and support needs, the iCBT program could be supplemented by therapist support. To keep the related costs of this guided format to a minimum, it is advisable to centralize the program within a limited number of treatment centers and have a relatively limited number of trained therapists. Future research is needed to be able to predict which women will benefit most from which format. Finally, as many BC survivors report a range of (interrelated) psychosocial and physical problems [49–51], we would recommend efforts to combine and integrate various iCBT interventions (e.g., for cancer-related fatigue, sleep problems, etc.) to better serve BC survivors and possibly reduce overall costs of psychosocial care in oncology settings.

### Limitations and strengths

This study has some limitations that should be noted. First, due to a lack of data, we did not include costs related to medication uptake. However, based on Mewes and colleagues [21], we expect these costs to be relatively low and similar across the intervention and control group. Second, we assessed healthcare consumption via generic questions that did not inquire specifically about the reason for utilization. It is likely that the differences in healthcare costs may not so much reflect the costs associated with the different formats of the iCBT program, but rather other factors. Third, there is increasing interest in conducting economic evaluations from a societal perspective, including costs associated with, among other things, productivity loss [52, 53]. While we had planned to include this perspective, it was evident to us that the productivity losses that were found during the trial could not be attributed to menopausal symptoms, but mainly to comorbid health conditions with which many BC survivors are faced.

This study also had noteworthy strengths. These included the RCT design, multicenter participation, high response rates, including both a healthcare and intervention perspective, evaluating both cost-utility, cost-effectiveness, and budget impact, and incorporating the intervention specific endpoints.

## Conclusion

This economic evaluation of guided and self-managed iCBT supports its cost-effectiveness in three respects. First, the cost-utility analysis indicates a cost per QALY well below frequently used thresholds. Second, the cost to obtain a clinically relevant reduction of menopausal symptoms and/or perceived impact of HF/NS is modest for both formats. Third, the budget impact of both programs is negligible when compared to the total healthcare expenditure for treating cancer in the Netherlands. Additionally, while treatment effects were only slightly greater in the guided format, the self-managed format was associated with substantially lower costs and more stable results when testing various assumptions and/or parameters in sensitivity analyses. Taken together, our results tend to favor the self-managed version of the iCBT program over the guided format, and thus we would favor a stepped care approach in which the self-managed version of the program is the default option, with the guided version being reserved for those situations where women have a strong preference for such support and where sufficient funding is available for the additional costs involved.

## Appendix A

Schematic representation of the model structure

### Abbreviations

#### Costs associated with health states

cMenSympt: costs of group with menopausal symptoms

cRed: costs of group with a clinically significant reduction

cRecuccernce1st: costs of Breast Cancer recurrence in the first year

cRecuccernce2nd*: costs of Breast Cancer recurrence in the second year

cDeath: costs of death

#### State change probabilities

pSympRedWithout: probability of a clinically significant reduction on FACT-ES for self-managed iCBT

pSympRedWith: probability of a clinically significant reduction on FACT-ES for guided iCBT

pSympRedWaitlist: probability of a clinically significant reduction on FACT-ES for waitlist control group

ToRec: probability of going to recurrence

GenMR: general mortality rate

MRBreastCancer: mortality rate breast cancer

#### Utilities

uMenSymp: utility for health state menopausal symptoms

uRed: utility for health state reduction in menopausal symptoms

uRecurrence: utility associated with the health state recurrence

uDeath: utility of the health state
